# Hemostasis-Modulating Proteins from Indian Elapid and Viper Snake Venom: Mechanistic Insights and Therapeutic Opportunities

**DOI:** 10.3390/life16101615

**Published:** 2026-09-26

**Authors:** Shanmugaraja Kiruthika, Aaron Varghese Sanju, Sameer Basha Hussain, Bipin Gopalakrishnan Nair, Muralidharan Vanuopadath

**Affiliations:** School of Biotechnology, Amrita Vishwa Vidyapeetham, Kollam 690525, Kerala, India

**Keywords:** snake venom, Indian snakes, elapids, viperids, hemostasis, platelet aggregation, coagulation

## Abstract

Snake venom is a complex cocktail of biologically active proteins and peptides with highly specific physiological activities, yet its therapeutic potential remains largely unexplored. India is home to several snake species, including vipers and elapids. Though viper venom is predominantly associated with hemostatic manifestations, several elapids can also disrupt hemostasis and cause venom-induced consumption coagulopathy. Snake venom protein families, including snake venom metalloproteases, serine proteases, phospholipases, disintegrins, snaclecs, L-amino acid oxidases, nucleotidases, and three-finger toxins, are involved in modulating the hemostatic system. In this review, we compile the available information on hemostasis-altering proteins from Indian elapids and vipers that have specific impacts on platelet aggregation and intrinsic, extrinsic, and common coagulation pathways. The molecular mechanisms of venom proteins in coagulation and platelet aggregation pathways are briefly discussed in relation to their functional consequences. Further, some of the venom proteins that are considered potential therapeutic leads targeting thrombotic disorders based on their mechanistic similarity with existing venom-derived and FDA-approved drugs are discussed. Overall, this review is focused not only on the mechanism of action behind the hemostatic-intervening proteins but also on their efficiency to develop novel therapeutics to treat thrombotic disorders.

## 1. Introduction

Venom has independently evolved over several hundred years across diverse animal lineages, from predatory groups such as snakes, scorpions, spiders, and cone snails to defensive and specialized organisms like jellyfish, sea anemones, centipedes, shrews, and vampire bats [1,2]. Among these, snake venoms are highly specialized biological secretions composed primarily of proteins and peptides along with other small molecules, including peptides, metal ions, and organic and inorganic constituents [3,4]. Snake venom proteins are broadly classified under enzymatic and non-enzymatic groups [5,6]. The enzymatic group is predominantly composed of protein families such as phospholipase A_2_ (PLA_2_), L-amino acid oxidases (LAAOs), snake venom metalloproteases (SVMPs), and serine proteases (SVSPs), and the non-enzymatic components include protein families such as three-finger toxins (3FTxs), disintegrins, and snaclecs [7,8]. Studies have shown that intra- and inter-species snake venom variation is influenced by many factors such as geographical distribution, age, gender, and diet [9,10,11,12]. While the clinical manifestations of envenomation vary significantly across snake families, in general, elapid venoms are neurotoxic and viper venoms are hemotoxic [13]. For example, venom from snake species representing elapids such as cobras and mambas contains a high proportion of proteins representing neurotoxic three-finger toxins, cytotoxins, and cardiotoxins. Neurotoxins disrupt the function of ion channels and receptors in the neuromuscular junction, resulting in paralysis and respiratory failure. Cardiotoxins affect the cardiovascular system by inducing muscle contraction and lysis of cardiomyocytes, which leads to myocardial damage and cardiac arrest [3]. Viper venom (true and pit vipers) is predominantly composed of protein families such as PLA_2_, metalloproteases, and serine proteases, which can cause hemorrhage, extensive tissue damage and disruption of hemostasis [14,15]. This target specificity in the course of evolution has made venoms excellent molecular templates for the development of drugs and therapeutics [16].

Studies have demonstrated that snake venom proteins possess diverse activities, including anticancer, antimicrobial, anti-inflammatory, and anticoagulant properties, making them valuable components for therapeutic applications [16,17,18,19,20,21]. Some FDA-approved drugs have successfully translated venom research into clinical practice, for example captopril (the antihypertensive from *Bothrops jararaca* Weid-Neuwied, 1824), eptifibatide, and tirofiban (based on echistatin from *Echis carinatus* Schneider, 1801), which are effective for the treatment of thromboembolic disease and acute myocardial infarction [22]. In addition, batroxobin has been used in the treatment of acute cerebral infarction, whereas hemocoagulase is used as a topical hemostatic in surgical interventions [23,24]. Some venom toxins also serve as essential diagnostic tools, such as Protac^®^ to measure protein C/S levels and RVV-X to detect lupus anticoagulants [25].

One of the key physiological activities found in viper venoms is their ability to modulate hemostasis by either inducing or inhibiting the blood coagulation pathway [26]. These actions can cause venom-induced consumption coagulopathy (VICC), which is characterized by the depletion of clotting factors and subsequent systemic hemorrhage [27]. As viper venoms are known to affect the hemostasis machinery, numerous studies have comprehensively described the diversity of viper and elapid snake venoms and their ability to interact with the human hemostatic system. Significant works on this aspect have provided detailed reviews on individual toxin families, including prothrombin activators, serine proteases disrupting fibrinolysis, and anticoagulant proteins and their clinical manifestations [27,28,29,30,31]. In this review, we compile and discuss the available information on proteins from Indian elapid and viper venoms affecting hemostasis, their possible molecular interactions within the hemostatic system, and emerging therapeutic applications of some of the key venom proteins from Indian snakes.

## 2. Snake Venom Proteins and Their Effects on Human Hemostasis

The hemostasis system is a regulated cascade mechanism that activates in response to hemorrhage. It is maintained by the balanced regulation of thrombogenic (clot-inducing) and antithrombogenic (clot-inhibiting) mechanisms [32]. It can be divided into two general steps. Primary hemostasis relies on the initial aggregation of platelets at the site of vascular injury. It is supported by secondary hemostasis involving sequential activation of coagulation factors through intrinsic and extrinsic coagulation pathways to form a solid fibrin clot [33].

Snake venom toxins intervene in this delicate physiological balance by modifying specific steps of the coagulation cascade with higher target specificity [17]. The most frequent clinical manifestation of these interventions is venom-induced consumption coagulopathy. A critical feature of many venom enzymes, particularly serine proteases found in snake venom, is their resistance to the body’s natural serine protease inhibitors (serpins), allowing them to evade normal physiological regulation. This results in continuous activation of clotting factors such as Factor X, Factor V, or prothrombin faster than the liver can replenish them [26,27]. This uncontrolled activity leads to the systemic depletion of essential factors, rendering the blood incoagulable and placing the victim at extreme risk for life-threatening, spontaneous hemorrhage. Understanding these specific molecular intervention points will help researchers develop more effective treatment strategies and engineer the next generation of highly specific antithrombotic therapeutics [31,34].

Major protein families involved in causing these effects include SVMPs, SVSPs, snaclecs, PLA_2_, disintegrins, LAAOs, nucleotidases, phosphodiesterases, and 3FTxs. Based on their molecular targets and structural characteristics, members of the same protein family may elicit procoagulant, anticoagulant, platelet-aggregating, or inhibitory activities. An overview of the reported hemostatic effects of these major venom protein families is presented in Table 1. Also, the list of proteins purified from Indian elapid and viper snake venoms and their reported mechanism of action are summarized in Table 2.

## 3. Venom Proteins from Indian Snakes Involved in Modulating Platelet Aggregation

Platelets are the main cellular responders in hemostasis. When subendothelial collagen is exposed due to vascular damage, von Willebrand factor (vWF) binds to the glycoprotein Ib-IX-V complex and interacts directly with GPVI and integrin α2β1 to promote platelet adhesion. This binding alters platelet shape and causes degranulation, resulting in the release of agonists such as ADP, serotonin, and thromboxane A_2_ to increase platelet aggregation. Platelet activation induces a conformational transition of integrin GPIIb/IIIa into a high-affinity state, enabling it to bind circulating fibrinogen or vWF. This leads to the cross-linking of adjacent platelets into a primary platelet plug, which is subsequently stabilized by a thrombin-mediated fibrin mesh and compacted by clot retraction [33,86,87].

Snake venoms have diverse toxins that block these processes with extreme specificity. These toxins disturb platelet function either by acting as agonists that induce aggregation, leading to systemic thrombocytopenia, or as antagonists that inhibit aggregation, thereby increasing the risk of massive hemorrhage [31,43]. These effects involve several high-affinity molecular targets, including the vWF receptor complex (GPIb-IX-V), the collagen receptors (GPVI and integrin α_2_β_1_), the fibrinogen receptor (integrin α_IIb_β_3_), and G-protein-coupled protease-activated receptors such as the purinergic receptor P2Y and protease activated receptors (PARs) [43,49]. The following section compiles the proteins from Indian snake species that interfere with the platelet aggregation process.

### 3.1. Snake Venom Proteins Involved in Inducing Platelet Aggregation

There are several venom proteins that indirectly induce aggregation by generating excess thrombin. Prothrombin activators such as Ecarin, Rusviprotease, and Trimarin convert prothrombin to thrombin, producing excessive thrombin that then activates platelet PARs [36,71,78]. Similarly, proteins such as RVV-X, RVBCMP, RV-FVP, and RVV-V are activators of Factors X and V, which promote thrombin formation that leads to platelet activation and aggregation [57,58,59,60]. Also, the same pattern is observed in malabarin from *T. malabaricus*, a protein that acts like thrombin by directly hydrolyzing fibrinogen subunits, thereby inducing platelet aggregation [77].

### 3.2. Snake Venom Proteins Involved in Inhibiting Platelet Aggregation

Several proteins from viper venoms inhibit the platelet aggregation process. For instance, there are snake venom proteins that directly influence the major receptors responsible for platelet plug formation. Echistatin is a potent disintegrin that binds with high affinity to the glycoprotein IIb/IIIa (GPIIb/IIIa) receptor with the help of its Arg-Gly-Asp (RGD) motif. This results in the blocking of fibrinogen binding, followed by prevention of platelet plug formation [74]. Another protein named echicetin specifically binds to GPIb, blocking its interaction with vWF and thereby disrupting the initial adhesion of platelets to damaged vessels [73]. NN-PF3, isolated from the Indian cobra (*N. naja*), is a high-molecular-mass metalloprotease that binds directly to the α2β1 integrin and further cleaves the GPVI receptor. This impairs platelet recognition of exposed collagen at the site of vascular injury [51]. L-amino acid oxidases such as BCV-LAO (*B. caeruleus*), K-LAO (*N*. *kaouthia*), and DrLAO (*D. russelii*) produce hydrogen peroxide, which retards the interaction between the GPIIb/IIIa receptor and fibrinogen, thereby preventing platelet activation [67,68,83].

Venom proteins can also inhibit platelet aggregation by interfering with extracellular agonists. The signaling molecules necessary for platelet aggregation, such as ADP and collagen, are also blocked by antagonists from snake venom. A phosphodiesterase, DR-PDE, from Russell’s viper (*D. russelii*) hydrolyzes the extracellular ADP, which is a vital recruitment signal utilized by platelets [66]. Additionally, phospholipase A_2_ isoenzymes from *N. naja* venom (NN-12c, NN-12d, and NN-12e) and EC-I-PLA_2_ from *E. carinatus* inhibit ADP-, collagen-, and epinephrine-induced platelet aggregation [56,75]. Similarly, KT-6.9, purified from the venom of *N. kaouthia*, acts as an antagonist that competitively binds the P2Y receptors on the platelet surface, thereby effectively blocking the binding of the ADP, thrombin, and arachidonic acid essential for the initiation of the clumping process [80]. Also, the blockage of thrombin is notable as it inactivates the PARs on the platelet surface, and this characteristic is exhibited by Daboxin P and Ruviprase from *D. russelii* and also Nk-PLA_2_β (*N. kaouthia*) [62,63,81]. A unique deaggregation property is shown by NnPLA_2_-I from the spectacled cobra, which hydrolyzes plasma phospholipids such as phosphatidylcholine into lysophospholipids. These byproducts disrupt existing platelet clumps and block further collagen-induced aggregation [55] (Figure 1).

## 4. Venom Proteins from Indian Snakes Involved in Anticoagulant Mechanisms

Endogenous anticoagulant systems, such as antithrombin, the tissue factor pathway inhibitor, and the protein C-protein S system, strictly regulate the physiological coagulation system. Snake venom proteins can interfere with these regulatory checkpoints by specifically targeting clotting factors, cofactors, phospholipids, or fibrinogen [32,88,89]. The identified anticoagulant proteins from Indian elapid and viper venoms are compiled in the following sections based on their primary sites of action.

### 4.1. Snake Venom Proteins Targeting the Intrinsic Coagulation Pathway

A low-molecular-weight serine protease, NnP28, from *N. naja* specifically prolongs the aPTT and recalcification time of human plasma while having negligible effects on the extrinsic pathway. Functional characterization also reveals that NnP28 targets an intrinsic pathway to retard clot formation [52]. Similarly, Rv(i)PLA_2_, purified from the Indian Russell’s viper (*D. russelii*), acts as a strong anticoagulant that significantly delays both the aPTT and recalcification time. Its primary effect is exhibited upstream of the common pathway, indicating specific interference with the early stages of the intrinsic cascade [65]. Similarly, a three-finger toxin isolated from the monocled cobra (*N. kaouthia*), Nk-3FTx, has been shown to exhibit mild anticoagulant effects. Unlike other neurotoxins, this protein induces a slight delay in the aPTT of human plasma, suggesting direct interference with the intrinsic coagulation cascade [82].

Studies using specific inhibitors like vanillic acid and concanavalin-A have established that 5′-nucleotidase from *N. naja* interferes directly or indirectly with one or more factors of the intrinsic pathway to delay blood coagulation. This enzyme has been identified as a more potent inhibitor of intrinsic hemostasis than previously characterized proteases in some cobra populations [54]. Novel anticoagulant isoforms purified from *B. fasciatus*, such as BF-AC1 and BF-AC2, elicit potent inhibitory effects on the early stages of the cascade. These heterodimeric proteins primarily target the extrinsic pathway but also inhibit the intrinsic tenase complex (Factor IXa and Factor VIIIa), with an IC_50_ of approximately 100 nM [84]. Similarly, Daboxin P is a major PLA_2_ enzyme that also acts as an inhibitor of FX/FXa and the intrinsic tenase complex. It disrupts the assembly of coagulation factors either by enzymatic hydrolysis of phospholipids or by non-enzymatically interfering with the stability of the complex [61].

### 4.2. Snake Venom Proteins Targeting the Extrinsic Coagulation Pathway

BF-AC1 and BF-AC2 are heterodimers found in the venom of *B*. *fasciatus*, consisting of a larger PLA_2_ subunit and a smaller kunitz-type serine protease inhibitor that are covalently linked by disulfide bonds, and they are isoforms of β-bungarotoxin. By hydrolyzing the essential phospholipids required for the assembly of the extrinsic tenase complex, these proteins inhibit FVIIa/tissue factor-mediated activation of Factor X. Their inhibitory action is strictly dependent on the enzymatic activity of the PLA_2_ subunit [84]. Another potent inhibitor of the extrinsic tenase complex is Daboxin P. It works enzymatically by cleaving phospholipids needed for complex assembly and prevents extrinsic tenase-mediated Factor-X-to-Xa conversion, which prevents it from non-enzymatically binding to essential factors in the cascade [61].

### 4.3. Snake Venom Proteins Acting Through a Common Pathway

The inhibition of FX and its active form, FXa, represents the most significant way to block the common pathway before thrombin burst occurs. The major PLA_2_ from *D. russelii*, named Daboxin P (~24% of crude venom), is a potent inhibitor of the common pathway. It targets both FX and FXa non-enzymatically by binding to the heavy chain of FXa that prevents the formation of the prothrombinase complex [61]. Daboxin P also binds and blocks thrombin [62]. In the same way, a peptide with a mass of 4.4 kDa isolated from the Eastern Indian Russell’s viper (*D. russelii*) venom is the dual inhibitor of FXa and thrombin. Unlike larger proteins, Ruviprase uses a non-enzymatic mechanism to directly inhibit FXa-mediated prothrombin activation and thrombin’s own catalytic activity, exhibiting slow-binding kinetics [63]. Similarly, Nn-PLA_2_-I purified from *N. naja* inhibits the common pathway by allosterically increasing the amidolytic activity of FXa while simultaneously degrading its ability to activate prothrombin, thereby preventing thrombin formation [55]. Studies have shown that Nk-PLA_2_α, isolated from *N. kaouthia*, exerts potent, non-enzymatic, and phospholipid-independent inhibition of FXa [81].

A stable conversion of prothrombin to thrombin requires the assembly of the prothrombinase complex (FXa, FVa, calcium, and phospholipids). Reports have shown that Neupholipase, purified from *D. russelii*, exerts its anticoagulant activity primarily by degrading phosphatidylserine, which is the essential phospholipid for prothrombinase assembly [64]. The basic PLA_2_ Rv(i)PLA_2_ also delays coagulation through effects on the common pathway. While it primarily acts upstream, its enzymatic hydrolysis of plasma phospholipids significantly affects the entire cascade [65]. The isoenzyme Nk-PLA_2_β from *N. kaouthia* is one of the most potent thrombin inhibitors, utilizing an uncompetitive mechanism to block the final stages of coagulation [81]. Similarly, NK-PLA_2_-I from the same species is a non-toxic, acidic anticoagulant phospholipase A_2_, that exerts its effect by prolonging the plasma recalcification time. It is involved in the hydrolysis of phospholipids, which is essential for the assembly of the prothrombinase complex, thereby preventing clot formation [30].

The final stage of the common pathway is the conversion of fibrinogen to a stable fibrin clot. Proteins that denature fibrinogen can render the blood incoagulable. Erythrofibrase, isolated from *T. erythrurus*, is a serine protease that acts as an α-fibrinogenase. It cleaves the Aα chain of fibrinogen, causing substantial cleavage of fibrin monomers and leading to hypofibrinogenemia that persists for days [76]. Similarly, a high-molecular-mass metalloproteinase, NN-PF3, inhibits the final step of clotting by directly degrading fibrinogen, effectively preventing thrombus formation [51]. Also, intravenous administration of Rusviprotease into mice results in massive in vivo defibrinogenation, rendering the blood incoagulable and leading to consumption coagulopathy [36]. Malabarase, purified from *T. malabaricus*, is a serine protease that specifically degrades the Aα and Bβ subunits of human fibrinogen. Unlike thrombin-like enzymes, malabarase does not release fibrinopeptides, but it makes the fibrinogen uncoagulable and significantly prolongs the plasma clotting times [79]. The venom possesses a unique sPLA_2_ isoform with a cationic anticoagulant loop. The three free basic residues (Arg 62, Lys 64, and Lys 66) in this loop form a cationic amphiphilic surface that interacts with negatively charged phospholipids, resulting in the blockage of multiple common pathway factors [85]. NnP28, an unusual 27.12 kDa serine protease from the Indian cobra, exerts strong anticoagulant and fibrinogenolytic activity. It prolongs aPTT and recalcification time by cleaving fibrinogen polymers, thereby preventing the formation of a thrombus [52]. Trimeric PLA_2_ isoforms and KPLA_2_ from *B. caeruleus* target the entire coagulation cascade through phospholipid depletion, which is essential for the assembly and formation of factors [69,70] (Figure 2a).

## 5. Venom Proteins from Indian Snakes Involved in Procoagulant Mechanisms

The procoagulant system comprises a set of clotting factors that primarily circulate in blood as inactive zymogens derived mainly from the liver [32]. The extrinsic pathway (tissue factor pathway) is the initial step that activates in vivo blood coagulation when tissue factor is released by injured tissues and binds to Factor VIIa to activate Factors X and IX [90]. The intrinsic pathway and the contact activation pathway involve the activation of Factor XII in the presence of negatively charged surfaces, such as subendothelial collagen. This triggers a sequence activating Factors XI, IX, and VIII. Both pathways converge on Factor Xa, which is then supported by binding to its cofactor Factor Va, calcium, and phospholipids to form the prothrombinase complex. Prothrombin is then converted to thrombin, which, in turn, cleaves fibrinogen to fibrin monomers that polymerize and undergo Factor XIII-mediated cross-linking to generate a stable, insoluble clot [91].

Snake venoms disrupt this cascade by deploying specialized procoagulant proteins that accelerate clotting factors, leading to uncoordinated clot formation and venom induced consumption coagulopathy [31].

### 5.1. Snake Venom Proteins Targeting the Intrinsic Coagulation Pathway

Snake venom toxins targeting upstream intrinsic factors accelerate the initial stages of coagulation. EC-III, a novel metalloproteinase purified from *E. carinatus*, facilitates procoagulant activity by reducing the recalcification time of human plasma. In vitro assays showed that EC-III reduced the normal clotting time by approximately two-fold [72]. Another metalloprotease called Trimarin from the venom of *T. malabaricus* shortens both the recalcification time and aPTT of human plasma by targeting the upstream factors of the coagulation pathway [78].

### 5.2. Snake Venom Proteins Targeting the Common Pathway

The initiation of the common pathway is most frequently targeted by the activation of Factors X and V that accelerate the physiological complexes. Russell’s viper (*D. russelii*) utilizes two major enzymes, namely RVV-X (metalloprotease) and RVV-V (serine protease), which specifically activate Factor X and Factor V, respectively. Also, the RVBCMP isolated from the same species is a 15 kDa basic metalloprotease that shortens the recalcification time and prothrombin time of plasma by activating FX or subsequent factors. This simultaneous activation forces the rapid assembly of the prothrombinase complex [58,59,60]. Another purified metalloprotease named Trimarin from *T. malabaricus* exhibits potent Factor Xa-like activity. It elicits clot formation in Factor X-deficient human plasma by proteolytically converting prothrombin to meizothrombin without the interference of Factor Xa [78]. Four novel serine protease isoenzymes from *D. russelii* venom (RV-FVP) induce blood coagulation specifically through FV activation, facilitating an increase in the rate of prothrombin activation when FXa is present [57].

The direct conversion of prothrombin to thrombin speeds up the entire coagulation cascade. An example of such action is seen with Ecarin, a class P-III metalloprotease and a newly identified Rusviprotease that convert prothrombin to meizothrombin, ensuring clot formation even in the absence of cofactors like Ca, phospholipids, or FV. This activity contributes to the venom-induced consumption coagulopathy seen in bite victims [36,71]. Malabarin, a major zinc-containing metalloprotease, shows potent thrombin-like activity. It acts directly at the terminal step of the cascade by hydrolyzing human fibrinogen to form a stable fibrin clot and releases fibrinopeptides A and B. Unlike typical fibrinogenases that render the molecule uncoagulable, malabarin mimics the proteolytic activity of thrombin to induce rapid, systemic clotting [77] (Figure 2b).

The purified hemostasis-altering proteins identified from Indian elapid and viper venoms that are described in the above sections are summarized with respect to their mechanisms of action in Table 3.

## 6. Venom-Derived Biotherapeutics: Current Advances and Future Perspectives

Animal venoms are considered natural mini bio-libraries where each molecule is pharmacologically active and evolved with higher specificity towards the target [2]. Specifically, snake venom is rich in proteins, peptides, and enzymes that target typical physiological barriers common in diseases associated with cardiovascular and neuromuscular systems [37]. Since snake envenomation can cause life-threatening conditions and given the limitations of currently available antivenoms, research focuses on improving treatment outcomes in envenomed patients and aims to reduce the morbidity and mortality rates. Nevertheless, several drugs derived directly from snake venom components have received approval from the FDA, primarily for treating hemostatic and cardiovascular conditions, such as captopril, tirofiban, integrilin, hemocoagulase, and batroxobin (defibrase) [23,24].

Aggrastat (tirofiban) is an antiplatelet drug designed based on echistatin (a disintegrin) isolated from the species *E. carinatus*. It is a reversible antagonist of the glycoprotein IIb/IIIa receptor (binds via the RGD motif), which prevents the binding of fibrinogen and subsequent platelet aggregation to manage acute coronary disorders and heart attacks [92]. Eptifibatide (integrilin) is a synthetic cyclic heptapeptide formulated based on barbourin, a disintegrin found in the venom of *Sistrurus miliarius barbouri* Gloyd, 1935. It targets the GPIIb/IIIa receptor via a highly specific KGD motif, effectively blocking the common pathway of platelet aggregation in patients with unstable angina or those undergoing percutaneous coronary intervention [93]. Exanta (Ximelagatran) is an orally administered anticoagulant that functions primarily as a direct thrombin inhibitor [94]. Hemocoagulase is also an FDA-approved drug used as a topical hemostatic agent. It is produced from *Bothrops* venom and promotes localized clotting by producing fibrin monomers and activating FX at the site of vascular injury or gastrointestinal bleeding [23]. Among serine proteases of therapeutic potential, defibrase/reptilase (batroxobin) is notable. Although the United States has not approved the drug clinically, other countries have. Batroxobin is a thrombin-like serine proteinase isolated from *Bothrops atrox* Linnaeus, 1758, and *Bothrops moojeni* Hoge, 1966. It catalyzes the release of fibrinopeptide A from fibrinogen, resulting in fibrin generation. As a procoagulant drug, it is utilized in the treatment of stroke, pulmonary embolism, deep vein thrombosis, myocardial infarction, and perioperative bleeding [17], [95]. Beyond serine proteases, other notable venom protein classes of therapeutic potential include botrocetin (a snaclec), a platelet-aggregating protein from the venom of *B. jararaca*. It enhances the affinity of the von Willebrand factor A1 domain for the platelet receptor glycoprotein Ibα [96].

Studies have also indicated that there are some venom-derived compounds that showed potent preclinical efficacy but failed in clinical trials. For example, Ancrod, isolated from the Malayan pit viper, is a thrombin-like enzyme that releases fibrinopeptide A to cause hypofibrinogenemia. Although it was used to treat stroke conditions, phase III clinical trials showed its inefficacy; hence, it was discontinued [97]. Similarly, Anfibatide, an antithrombotic agent, is a snaclec purified from the sharp-nosed viper (*Deinagkistrodon acutus* Gunther, 1888). It targets the GPIbα receptor on platelets by blocking its interaction with vWF and thrombin to prevent platelet adhesion and thrombus formation [8].

Our review identifies several Indian venom proteins that resemble the mechanism of clinically developed or approved drugs from snake venom, as summarized in Table 4. Similar to anfibatide, echicetin, a heterodimer from *E. carinatus*, specifically binds to GPIb to block platelet adhesion and vWF interaction. Its relatively long half-life in blood (~170 min) and higher specificity make it a promising and stable candidate for preventing arterial thrombosis [73]. Similarly, like batroxobin, malabarin also cleaves the Aα subunit followed by the Bβ subunit of fibrinogen, releasing fibrinopeptides A and B. Its intravenous administration causes systemic defibrinogenation, depleting circulating fibrinogen levels and rendering the blood incoagulable. As it is not inhibited by α2-macroglobulin, it can exert sustained systemic effects and could represent a potential lead for the therapeutic removal of occlusive thrombin [77]. Similarly, malabarase also degrades the Aα and Bβ subunits of fibrinogen. However, it lacks the ability to release fibrinopeptides. This property makes the fibrinogen uncoagulable without the risk of forming initial microthrombi, a common side effect of traditional snake venom thrombin-like enzymes (SVTLEs). Notably, malabarase is also non-toxic and non-hemorrhagic and cannot degrade extracellular matrix proteins like collagen or fibronectin, making it a potential candidate for treating thrombotic disorders [79]. Unlike the other primary toxic components of cobra venom (like 3FTx/PLA_2_s), NN-PF3 is non-lethal and functions through selective fibrinogenolysis independently of cofactor requirements. This provides a unique avenue for developing selective coagulation inhibitors with reduced systemic toxicity [50]. RVBCMP is a Factor X activator and also acts as a specific α-fibrinogenase. Its procoagulant action makes it a possible template for developing a topical hemostatic agent like hemocoagulase to manage localized bleeding. Structural analysis of RVBCMP reveals that it possesses distinct sites for coagulant and hemorrhagic activities, suggesting that it can be further screened to explore its pro-hemostatic potential by reducing its local tissue-damaging effects [60].

## 7. Conclusions

Indian elapid and viper venoms consist of diverse peptides and proteins that interfere with hemostasis through unique and often highly specific mechanisms. Several purified proteins are known to induce or inhibit platelet aggregation, coagulation factor activation, thrombin generation, utilization of fibrinogen, and fibrin formation or degradation. Also, the crude venom of some species was found to be a procoagulant by activating prothrombin (*N. naja*) [53] and reducing plasma recalcification time, aPTT, and PT (*N. naja*, *Ophiophagus hannah* Cantor, 1836) [58,98]. Crude venoms from certain species have been shown to exert anticoagulant activity by causing fibrinogenolytic activity and prolonging PT and aPTT [99]. Despite these findings, many hemostasis-altering proteins with potent procoagulant and anticoagulant properties remain poorly characterized, particularly with respect to their purification and molecular characterization. Snakebite-mediated tissue injury may cause the activation of the extrinsic pathway; however, the venom-induced procoagulant effects remain poorly understood. Notably, the mechanistic similarity of these proteins with existing snake venom-derived drugs has been explored, which supports the feasibility of utilizing them as a template for targeted drug development especially focused on thrombotic disorders. Future studies should focus more on the purification and characterization of hemostasis-modulating proteins followed by the elucidation of their molecular targets and structure–function relationship from Indian elapid and viper venoms. Nevertheless, therapeutic translation will require establishing target selectivity, efficacy, safety, reproducibility, and pharmacokinetic properties through in vivo models, followed by several phases of clinical trials. The integration of venomics with functional characterization and targeted biochemical assays may help in the identification of novel antithrombotic molecules. Overall, the systematic characterization of Indian snake venom proteins may facilitate the identification of therapeutically valuable drug candidates that could be potentially translated to clinical settings.

## Figures and Tables

**Figure 1:**
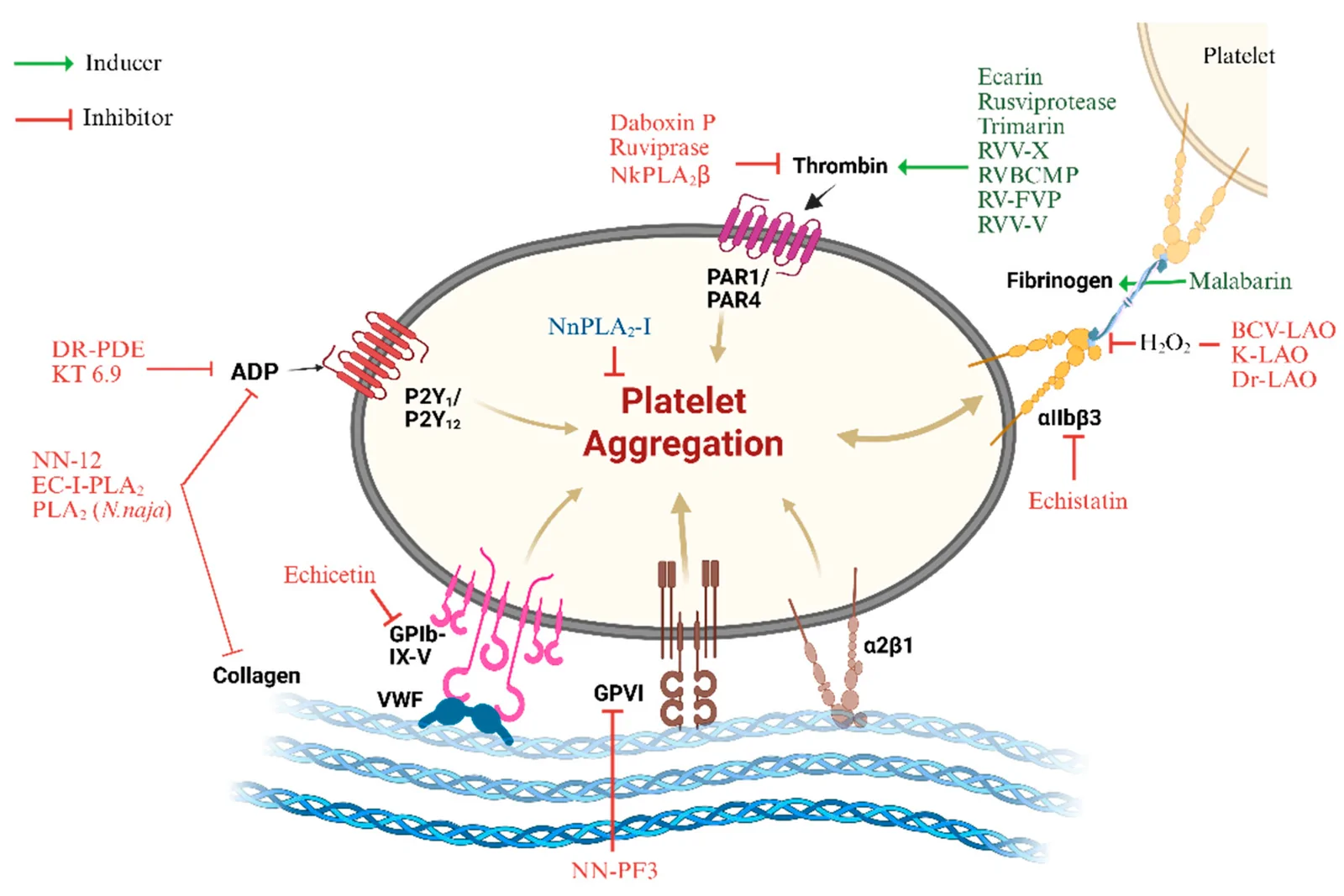
Platelet activators and inhibitors purified from Indian snake venom. PAR, protease-activating receptor; P2Y1/P2Y12, purinergic receptor; GPIb-IX-V, glycoprotein Ib-IX-V receptor complex; GPVI, glycoprotein VI receptor; α2β1, α2β1 receptor; αIIbβ3, αIIbβ3 receptor.

**Figure 2:**
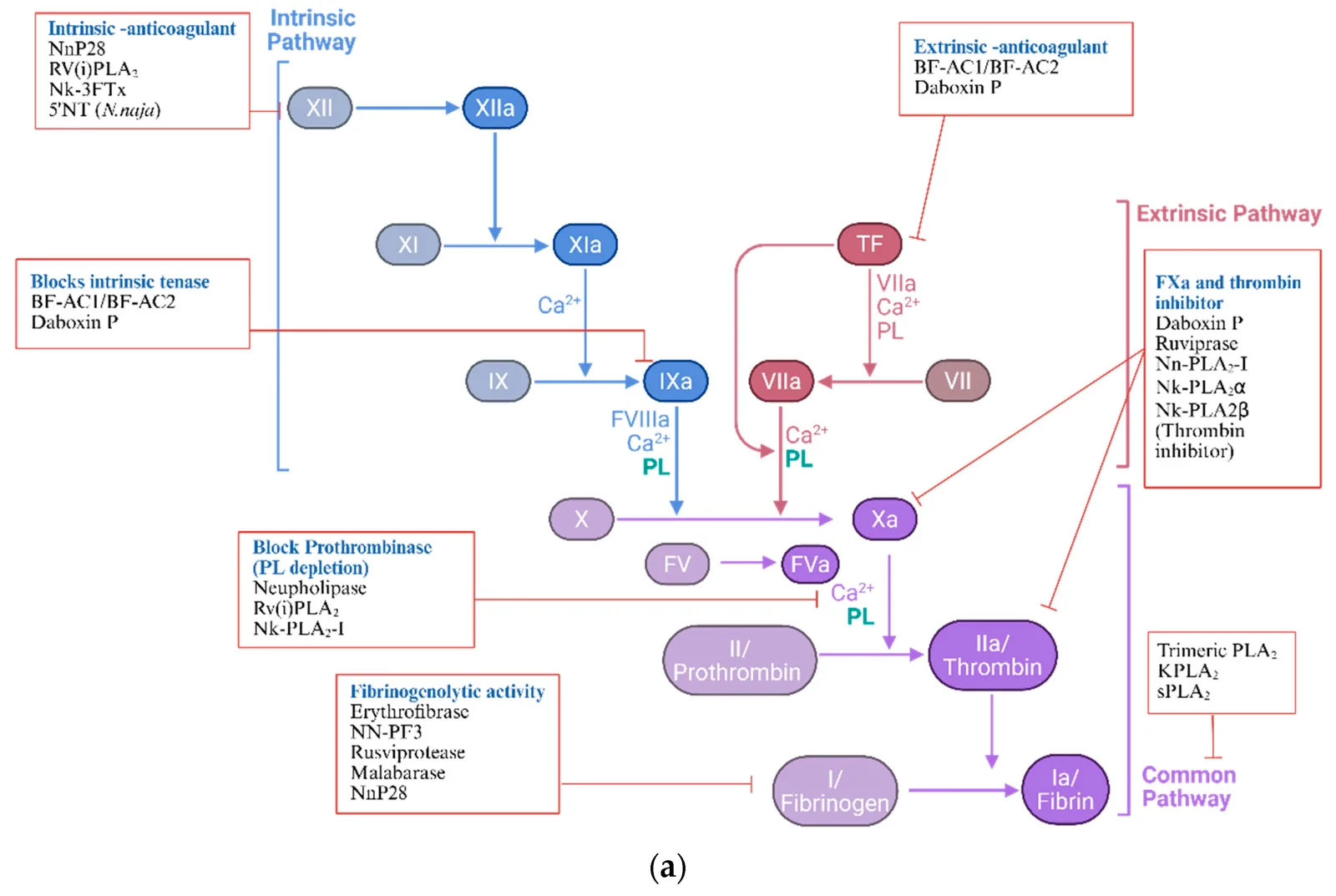

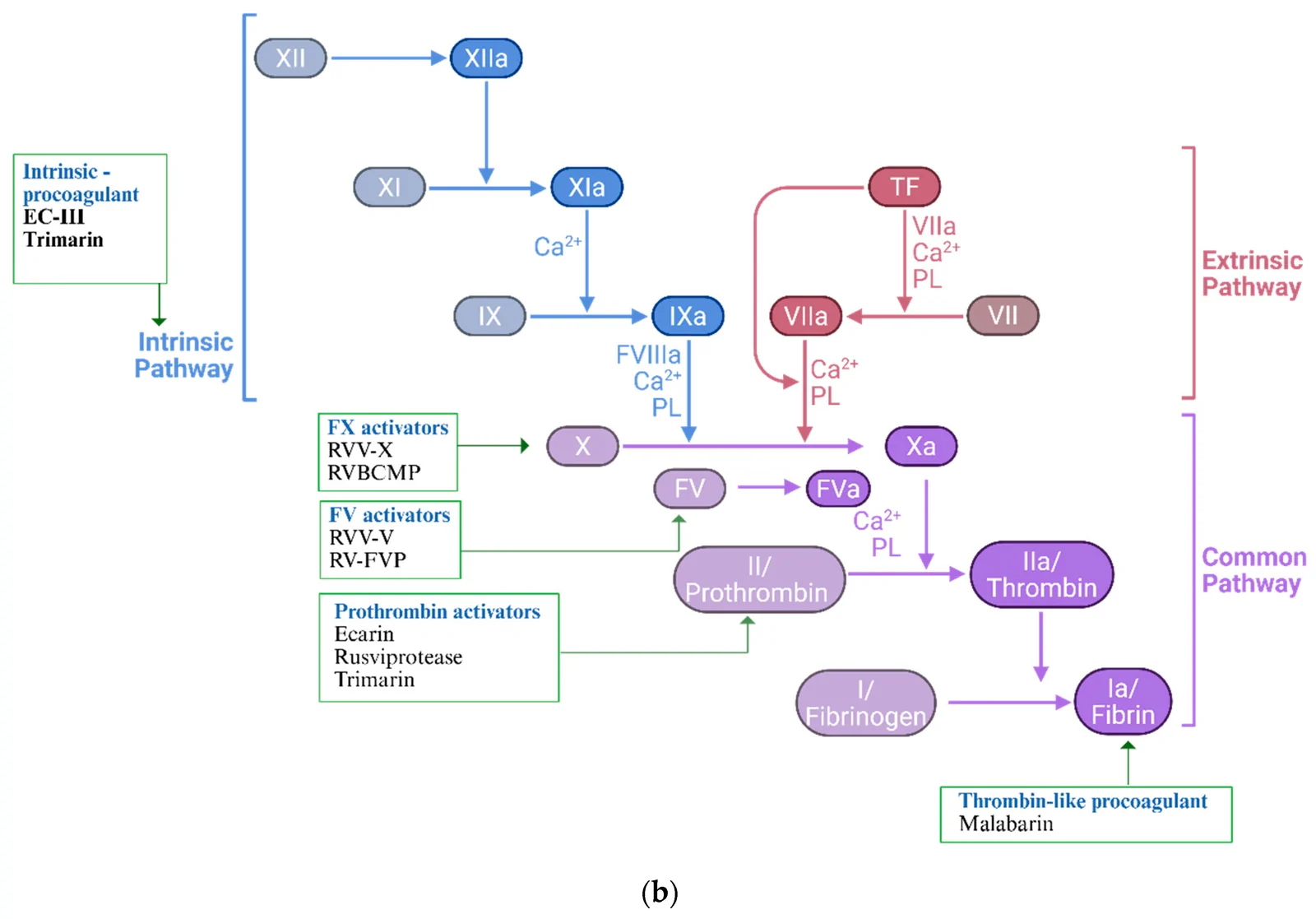
Purified proteins involved in modulation of intrinsic, extrinsic and common pathways. (**a**) Anticoagulant and (**b**) procoagulant proteins purified from Indian snake species. The figure illustrates venom proteins that activate or inhibit specific factors and components involved in the coagulation pathway. PL, phospholipid.

**Table 1:** Major snake venom protein families involved in altering the human hemostatic system.

Mode of Action		Procoagulant Activity	Anticoagulant Activity	Platelet Aggregation Inhibition Activity	Platelet Aggregation Inducing Activity	References
	Snake Venom Protein Family					
Snake venom metalloproteinases		✓	✓	✓	✓	[35,36]
Snaclecs		✓	✓	✓	✓	[29,31,37]
Snake venom serine proteases		✓	✓	✗	✓	[38,39,40]
L-amino acid oxidases		✗	✓	✓	✓	[8,41]
Disintegrins		✗	✗	✓	✗	[22,42]
Phospholipases (PLA_2_)		✗	✓	✓	✓	[27,43,44]
Nucleotidases and phosphodiesterases		✗	✓	✓	✗	[45,46,47,48]
Three-finger toxins		✗	✓	✓	✗	[29,49]

✓, activity reported; ✗, activity not reported.

**Table 2:** Venom proteins purified from Indian vipers and elapids involved in altering human hemostasis.

SI.No	Protein Name	Possible Mechanism of Action	Inference from the Study	Protein Family	Reference
*Naja naja* Linnaeus, 1758					
1.	NN-PF3	Anticoagulant—common	Acts as a fibrinogenase, degrading the Aα subunit and then the Bβ subunit of fibrinogen. The protein showed a strong anticoagulant property in a dose- and time-dependent manner.	SVMP	[50]
2.	NN-PF3	Platelet aggregation inhibitor	Inhibits collagen-induced aggregation by binding to the α2β1 integrin and interferes with the GPVI signaling pathway.	SVMP	[51]
3.	NnP28	Anticoagulant—common and intrinsic	Elicits fibrinogenolytic activity by degrading the Aα subunit of fibrinogen; also, it prolongs recalcification time and aPTT.	SVSP	[52]
4.	PLA_2_	Platelet aggregation inhibitor	Inhibits ADP- and collagen-induced platelet aggregation	PLA_2_	[53]
5.	5′-nucleotidase	Anticoagulant—intrinsic	It interacts directly/indirectly with one or more factors of the intrinsic pathway to delay blood coagulation.	5′-NT	[54]
6.	NNPLA_2_-I	Anticoagulant—common and a platelet aggregation inhibitor	It inhibits thrombin formation in a non-enzymatic manner and also thrombin-induced platelet aggregation.	PLA_2_	[55]
7.	(NN-12c, NN-12d, NN-12e)	Platelet aggregation inhibitor	It inhibits platelet aggregation induced by ADP, collagen, and epinephrine by catalytic activity through modification of the platelet surface.	PLA_2_	[56]
*Daboia russelii* Shaw and Nodder, 1797					
8.	Rusviprotease	Procoagulant—common; anticoagulant—common	Acts as a group A prothrombin activator and converts prothrombin to thrombin independent of cofactors. The protein also exhibited defibrinogenlytic activity in in vivo studies.	SVMP	[36]
		Platelet aggregation inducer	It produces excess thrombin, which binds the PAR and induces aggregation.		
9.	RV-FVP	Procoagulant—common and a platelet aggregation inducer	The purified proteins act as Factor V activators and induce blood coagulation (in vitro), which increases thrombin generation and causes defibrinogenation (in vivo).	SVSP	[57]
10.	RVV-X	Procoagulant—common and a platelet aggregation inducer	It is a Factor X activator and initiates the common pathway by FX-to-FXa conversion, thereby promoting clot formation.	SVMP	[58]
11.	RVV-V	Procoagulant—common and a platelet aggregation inducer	It activates Factor V by selective cleavage at site III (Arg1545-Ser1546).	SVSP	[59]
12.	RVBCMP	Procoagulant—common and a platelet aggregation inducer	It shortens the recalcification time and PT by activating FX.	SVMP	[60]
13.	Daboxin P	Anticoagulant—intrinsic, extrinsic, and common	Inhibits conversion of Factor X to Xa. It acts as an inhibitor of tenase complexes (intrinsic and extrinsic) and prothrombinase (common) by binding to FX and Fxa.	PLA_2_	[61]
14.	Daboxin P	Platelet aggregation inhibitor	It interacts with thrombin, inhibits platelet formation by blocking PARs, inhibits collagen-induced and not ADP- or arachidonic acid-induced platelet aggregation, and inhibits thrombin-induced platelet aggregation.	PLA_2_	[62]
15.	Ruviprase	Anticoagulant—common	Dual inhibitor of thrombin and Factor Xa via non-enzymatic binding.	4 kDa peptide	[63]
		Platelet aggregation inhibitor	It inhibits thrombin-induced platelet aggregation by blocking the thrombin required to cleave PARs.		
16.	Neupholipase	Anticoagulant—common	It preferentially binds to and hydrolyzes phosphatidylserine, phosphatidylcholine, and phosphatidylethanolamine, which are essential for assembly of the prothrombinase complex.	PLA_2_	[64]
17.	Rv(i) PLA_2_	Anticoagulant—intrinsic and common	It prolongs the recalcification time and APTT. It degrades the phospholipids required for the assembly of factors.	PLA_2_	[65]
18.	DR-PDE	Platelet aggregation inhibitor	It enzymatically binds and hydrolyzes extracellular ADP, which is an agonist vital for platelet plug formation (inhibits ADP-induced platelet aggregation).	PDE	[66]
19.	DrLAO	Platelet aggregation inhibitor	Excess H_2_O_2_ reduces the interaction between fibrinogen and activated integrin GPIIb/IIIa, thus inhibiting platelet aggregation. Inhibits ADP- and collagen-induced platelet aggregation.	LAAO	[67]
*Bungarus caeruleus* Schneider, 1801					
20.	BCV-LAO	Platelet aggregation inhibitor	Excess H_2_O_2_ reduces ADP binding or interferes with the interaction between fibrinogen and integrin αIIbβ_3_.	LAAO	[68]
21.	KPLA_2_	Anticoagulant—common	Enzymatic depletion of phospholipids, which are the essential cofactors for the formation of fibrin clots.	PLA_2_	[69]
22.	Trimeric PLA_2_ isoform	Anticoagulant—common	Trimeric PLA_2_ isoforms are more stable than monomers and contribute to overall anticoagulant activity.	PLA_2_	[70]
*E. carinatus*					
23.	Ecarin	Procoagulant—common	It acts as a single-chain prothrombin activator that converts prothrombin to thrombin independently of Ca^2+^, phospholipids, and Factor Va.	SVMP	[71]
		Indirect platelet aggregation inducer	Generates thrombin—a potent platelet agonist.		
24.	EC-III	Procoagulant—intrinsic	Reduced recalcification time in human plasma. It possesses proteolytic activity but is non-hemorrhagic and non-myotoxic.	SVMP	[72]
25.	Echicetin	Platelet aggregation inhibitor	A heterodimer binds to the platelet GPIb receptor. This binding blocks its interaction with vWF and platelets, preventing primary adhesion and agglutination.	C-type lectin	[73]
26.	Echistatin	Platelet aggregation inhibitor	It binds with integrin GPIIb/IIIa, competitively inhibiting fibrinogen binding required for platelet aggregation.	Disintegrin	[74]
27.	EC-I-PLA_2_	Platelet aggregation inhibitor	Dose- and time-dependent inhibition of platelet aggregation induced by ADP, collagen, and epinephrine.	PLA_2_	[75]
*Trimeresurus erythrurus* Cantor, 1839					
28.	Erythrofibrase	Anticoagulant—common	It shows direct α-fibrinogenase activity, degrading the Aα chain of fibrinogen.	SVTLEs	[76]
*Trimeresurus malabaricus* Jerdon, 1854					
29.	Malabarin	Procoagulant—common and a platelet aggregation inducer	Malabarin acts directly on fibrinogen to initiate clot formation by hydrolyzing the Aα subunit followed by the Bβ subunit of fibrinogen. This releases fibrinopeptide A and fibrinopeptide B, which resemble thrombin, to form a fibrin clot.	SVMP	[77]
30.	Trimarin	Procoagulant—common and an intrinsic, platelet aggregation inducer	Trimarin shortens APTT and PT. It functions as a Factor Xa-like activator/prothrombin activator, clotting Factor X-deficient plasma, by converting prothrombin to meizothrombin, which then converts fibrinogen to fibrin.	SVMP	[78]
31.	Malabarase	Anticoagulant—common	Malabarase selectively targets fibrinogen, hydrolyzing the A and B chains in a way that makes the fibrinogen uncoagulable, prolonging plasma coagulation time.	SVSP	[79]
*Naja kaouthia* Lesson, 1831					
32.	KT-6.9	Platelet aggregation inhibitor	It is a low-molecular-weight peptide that inhibits platelet aggregation induced by ADP, thrombin, and arachidonic acid. It acts as an antagonist by binding to GPCRs such as the P2Y receptor on the platelet surface, preventing agonist binding.	3FTx	[80]
33.	Nk-PLA_2_β	Anticoagulant—common	Non-enzymatic inhibition of thrombin, and it prevents the conversion of fibrinogen to a fibrin clot.	PLA_2_	[81]
		Platelet aggregation inhibitor	Partial inhibitor of thrombin-induced platelet aggregation.		
	Nk-PLA_2_α	Anticoagulant—common	It acts via non-enzymatic inhibition of FXa independently of phospholipids/calcium.		
34.	NK-PLA_2_-I	Anticoagulant—common	It prolongs plasma clotting time through enzymatic hydrolysis of phospholipids, which are essential for prothrombinase complex formation.	PLA_2_	[30]
35.	NK-3FTx	Anticoagulant—intrinsic	It exhibits mild anticoagulant activity by prolonging aPTT.	3FTx	[82]
36.	K-LAO	Platelet aggregation inhibitor	Generation of H_2_O_2_ inhibits platelet aggregation induced by ADP, collagen, and ristocetin in a dose-dependent manner.	LAAO	[83]
*Bungarus fasciatus* Schneider, 1801					
37.	BF-AC1	Anticoagulant—extrinsic and intrinsic tenase complexes	Both inhibit the extrinsic (cleave the phospholipid surface required for FVIIa and TF) and intrinsic (FIXa and VIIIa) tenase complexes through their PLA_2_ activity.	β-bungarotoxin	[84]
	BF-AC2				
*Naja sagittifera* Wall, 1913					
38.	sPLA_2_	Anticoagulant—common	It contains a specific anticoagulant loop with a positive charge that complexes with phospholipids, interfering with coagulation factors.	PLA_2_	[85]

**Table 3:** Venom protein families from Indian snake species involved in altering the hemostatic system.

				Anticoagulant			Procoagulant		
Mode of Action		Platelet Aggregation Activation	Platelet Aggregation Inhibition	Intrinsic	Extrinsic	Common	Intrinsic	Extrinsic	Common
	Snake Venom Protein Family								
Snaclecs		-	+	-	-	-	-	-	-
Snake venom metalloproteinases		+	+	-	-	+	+	-	+
Snake venom serine proteases		+	-	+	-	+	-	-	+
L-amino acid oxidases		-	+	-	-	-	-	-	-
Disintegrins		-	+	-	-	-	-	-	-
Phospholipases (PLA_2_)		-	+	+	+	+	-	-	-
5′-Nucleotidases		-	-	+	-	-	-	-	-
Phosphodiesterases		-	+	-	-	-	-	-	-
β-bungarotoxin		-	-	+	+	-	-	-	-
Three-finger toxins		-	+	+	-	-	-	-	-

**Table 4:** Clinically developed venom-derived drugs and putative alternatives from Indian snake venoms.

FDA-Approved/Clinically Developed Snake Venom-Derived Drugs and Their Mechanisms	Putative Alternatives from Indian Snakes	Snake Species	Reported Mechanisms of Action	References
Anfibatide: Blocks platelet GPIbα interaction with vWF and thrombin	Echicetin	*E. carinatus*	It binds to GPIb, inhibiting vWF interaction and preventing platelet adhesion.	[73]
Batroxobin: Releases fibrinopeptide A and induces defibrinogenation	Malabarin	*T. malabaricus*	It cleaves the Aα and Bβ subunits of fibrinogen, releasing fibrinopeptides A and B and causing systemic defibrinogenation.	[77]
	Malabarase	*T. malabaricus*	It cleaves the Aα and Bβ subunits of fibrinogen, without releasing fibrinopeptides A and B, potentially reducing microthrombus formation.	[79]
	NN-PF3	*N. naja*	It exhibits selective fibrinogenolysis without cofactor activity.	[50]
Hemocoagulase: Promotes fibrin formation and activates coagulation locally	RVBCMP	*D. russelii*	Its properties of FX activation and fibrinogenolytic activity suggest potential development as a localized pro-hemostatic agent.	[60]

## Data Availability

No new data were created or analyzed in this study. Data sharing is not applicable to this article.
